# Poor oral health and mortality in geriatric patients admitted to an acute hospital: an observational study

**DOI:** 10.1186/s12877-020-1429-z

**Published:** 2020-01-28

**Authors:** Keisuke Maeda, Naoharu Mori

**Affiliations:** 1 Department of Palliative and Supportive Medicine, Graduate School of Medicine, Aichi Medical University, 1-1 Yazakokarimata, Nagakute, Aichi 480-1195 Japan; 2 Department of Nutrition and Dysphagia Rehabilitation, Tamana Regional Health Medical Center, 2172 Tamana, Tamana, Kumamoto, 865-0005 Japan

**Keywords:** Inpatient, Oral health assessment, Survival

## Abstract

**Background:**

Poor oral health at hospital admission is a potential higher mortality risk predictor. We aimed to determine in-hospital mortality by assessing poor oral health using a validated tool.

**Methods:**

A retrospective observational study was conducted in an acute care hospital, and 624 consecutive geriatric patients were included. Patients were divided into three groups according to oral health, stratified by the Oral Health Assessment Tool (OHAT) scores. Nutritional status, daily living activities, cognitive impairment, and comorbidities were collected as covariates. Univariate and multivariate analyses were performed to identify the relationship between oral health and survival.

**Results:**

The mean age was 83.8 ± 7.9 years, and 41% were males. Groups with an OHAT score equivalent to 0, 1–2, and ≥ 3 comprised 213, 206, and 205 patients, and 11 (5.2%), 13 (6.3%), and 37 (18.0%) of those patients died in the hospital, respectively. Patients in the OHAT score ≥ 3 group had higher mortality than those in the other groups (log-rank test: *p* = 0.012 for the OHAT = 0 group; *p* = 0.010 for the OHAT = 1–2 group after Bonferroni corrections). Patients in the OHAT score ≥ 3 group continued to have poor survival even after adjusting for confounders in the Cox’s regression analysis (hazard ratio: 2.514, 95% confidence interval: 1.220–5.183, *p* = 0.012).

**Conclusion:**

In geriatric patients, poor oral health at hospital admission was an independent in-hospital mortality predictor. Future studies on oral care intervention stratified by oral health conditions are warranted.

## Background

Oral health is important for the maintenance of good overall health. In the field of geriatric medicine, poor oral health has been known to be associated with physiological burden [1], sarcopenia [2], cognitive impairment [3], and accumulating comorbidities [4]. Furthermore, poor oral health increases hospitalization risk due to both infectious [5] and non-infectious diseases [6]. Therefore, clinicians have recently started focusing on oral health care. Poor oral health also impacts mortality. A large community-based cohort study conducted over approximately 40 years revealed that the number of teeth, marginal born, and dental plaque are associated with all-cause mortality [7]. In addition, improvement in oral health reportedly has a positive influence on mortality. A systematic review demonstrated that the treatment of tooth loss protects against mortality [8], and another systematic review revealed that oral care intervention during hospitalization can result in more favorable mortality-related outcomes [9].

The risk of mortality owing to poor oral health in out-of-hospital settings have been extensively studied; however, to our knowledge, despite the existence of several studies on oral care intervention in a hospital setting, studies investigating the association between poor oral health and mortality within a hospital setting are lacking [9, 10]. Previously, we demonstrated the possible relationship between oral health at hospital admission and mortality in patients with aspiration pneumonia [11]. One major point of concern regarding the studies investigating the association between oral health and mortality is that most previous studies evaluated oral health only based on dental problems such as the number of teeth, existing periodontitis of the gum, or tooth caries; however, poor oral health can also be identified by considering multiple factors including the condition of the tongue, lips, and saliva. Recently, some indices for oral health were developed by considering multiple factors, and the reliability and validity of these indices were determined [12].

In this study, we aimed to investigate whether poor oral health as assessed by a validated tool could predict mortality and factors associated with poor oral health of geriatric in-patients at the time of hospital admission.

## Methods

### Design and participants

This retrospective observational study was conducted in a 53-bed acute medical ward of our hospital managed by a medical association in a city with a population of approximately 100,000, of which 30.7% are older adults (age ≥ 65 years). The study included ≥65-year-old patients who were admitted to the hospital for treatment between April and December of 2016. The exclusion criteria included patients whose oral health assessment (using the Oral Health Assessment Tool [OHAT]) [12] could not be performed until the day after admission and patients who declined to participate in the study. Because it was a retrospective study, an opt-out opportunity within two months was announced in the hospital during the time of recruitment, and patients and their families could withdraw from the study at any time. The study was approved by the Institutional Review Board and the Ethics Committee of Tamana Regional Health Medical Center, in accordance with the tenets of the Declaration of Helsinki (ID: TRHMC291225).

### Measurements

The patients’ data, including age, sex, nutritional status, activities of daily living (ADL), cognitive status, and comorbidities were collected from their medical records. All the items were routinely evaluated and recorded by trained nurses at hospital admission. Nutritional status was assessed using body mass index, which is calculated as body weight [kg] divided by height [m] squared, and the Mini-Nutritional Assessment Short Form (MNA-SF) [13], the score of which ranges from 0 to 14; the scores of 0–7, 8–11, and 12–14 indicate malnutrition, risk of malnutrition, and normal nutritional status, respectively. ADL prior to disease onset was assessed using the Barthel Index [14], which ranges from 0 (dependent) to 100 (independent) using a 5-point scale. Cognitive status was evaluated using the Cognitive Performance Scale [15], which ranges from 0 (intact) to 6 (very severe impairment) in a hierarchical scale created from five items associated with cognitive function. Degree of comorbid diseases was assessed using the Charlson Comorbidity Index [16]. The index, which consists of 19 comorbid conditions, is the sum of scores assigned based on the number and severity of various conditions. One point is assigned for the presence of chronic obstructive pulmonary disease, chronic heart failure, myocardial infarction, diabetes mellitus without complication, mild liver disease, peripheral vascular disease, cerebrovascular accident, transient ischemic attack, dementia, connective tissue disease, and peptic ulcer disease; two points are assigned for the presence of diabetes mellitus with end-organ damage, moderate to severe chronic kidney disease, hemiplegia, solid malignant tumor, leukemia, and lymphoma; three points are assigned for the presence of moderate to severe liver disease; and six points are assigned for the presence of metastatic solid tumor and acquired immunodeficiency syndrome. A higher summed score indicates a greater number of comorbidities associated with mortality.

Oral health was assessed using OHAT by dental hygienists immediately after admission. OHAT is a validated tool for the assessment of oral health, which comprises eight domains, including lips, tongue, gums and tissues, saliva, natural teeth, dentures, oral cleanliness, and dental pain, which are stratified into three grades (healthy, oral changes, or unhealthy). The scores of the eight domains are summed to create a total score, ranging from 0 (healthy) to 16 (unhealthy) [12]. For example, in the lips domain, smooth, pink, and moist indicates a healthy grade; dry, chapped and red at corners indicate oral changes; and swelling or lump, white/red/ulcerated patch, and bleeding/ulcerated at corners indicate an unhealthy grade. In the tongue domain, normal, moist roughness, and pink indicate a healthy grade; patchy, fissured, red, and coated indicate oral changes; and a patch that is red and/or white, ulcerated, and swollen indicates an unhealthy grade. In the gum and tissues domain, pink, moist, smooth, and no bleeding indicate a healthy grade; dry, shiny, rough, red, swollen, and one ulcer/sore spot under dentures indicate oral changes; and swollen, bleeding, ulcers, white/red patches, and generalized redness under dentures indicate an unhealthy grade. Details of the criteria for the other five domains in the OHAT assessment can be found in the original study [12]. The developers of the OHAT suggest that people identified as having any ‘changes’ or as ‘unhealthy’ in any subcategories of the OHAT should be examined by a dentist [12]. In the preliminary study conducted in the hospital before performing the current investigation, we noted that groups with an OHAT score of 0, 1–2, and ≥ 3 involved similar proportions of patients (unpublished). Furthermore, an OHAT score of 1–2 indicates that either the patient’s condition ‘changes’ in only one or two categories, or that the patient’s condition is ‘unhealthy’ in only one category while their condition in all other categories is ‘healthy.’ Likewise, an OHAT score of ≥3 indicates that the patient’s condition is not ‘healthy’ in at least two categories. Thus, in the current study, we classified patients into three groups based on the OHAT scores of 0, 1–2, and ≥ 3.

The outcome was considered to be in-hospital mortality within 60 days. The observational period was determined based on a previous study conducted in the hospital, indicating that the median length of stay in the hospital was 27 [interquartile range, 1750] days [17].

### Sample size calculation

In a previous study conducted in the hospital, 12% of hospitalized patients died in the hospital within 60 days [17]. If we assumed that patients belonging to the highest tertile of OHAT (poorest) would show a mortality ratio of 20% and the other patients would show a ratio of 9%, at least 583 participants would be required to reject the null hypothesis with a power of 0.9 and an alpha error of 0.05. Therefore, we planned a study period with sufficient duration to be able to include an appropriate number of participants.

### Statistical analysis

Quantitative variables are expressed as the mean ± standard deviation (parametric) or median [interquartile range] (non-parametric) based on each histogram. Differences between groups were analyzed using the analysis of variance (parametric) or Kruskal–Wallis test (non-parametric). Categorical data are expressed as the frequency (percentage), and differences were analyzed using the chi-square test. When there was a significant difference in the post-hoc multiple comparison analysis, Bonferroni’s correction was performed. The Kaplan–Meier curve was used to compare the probability of survival among patients stratified in the three OHAT groups, and differences were compared using the log-rank test. Cox’s regression analysis was performed to determine whether oral health could be an independent predictor of mortality after adjusting for variables such as length of hospital stay, age, sex, nutritional status, cognitive status, ADL, comorbidities, and reasons for hospital admission. Analyses were performed using SPSS 21.0 software (IBM Corp., Armonk, NY, USA), and a *p*-value of < 0.05 was considered to be statistically significant.

## Results

A total of 650 geriatric patients were admitted to the hospital during the study period. Of these, the oral health of 26 patients was not assessed within two days. None of the patients refused to participate in the retrospective study. Consequently, the study included 624 patients. The baseline characteristics of the subjects are summarized in Table 1. The mean age of these patients was 83.8 ± 7.9 years, and 40.7% were males. Approximately one-third of the subjects were considered to be malnourished based on the MNA-SF score, and over half of the subjects needed some assistance for ADL as per the assessment made using the Barthel Index score.

**Table 1:** Characteristics of studied subjects

Variables	All (*n* = 624)
Age, years, mean ± SD	83.8 ± 7.9
Male, n (%)	254 (40.7)
BMI, kg/m^2^, mean ± SD	20.4 ± 3.8
Nutrition	
Normal nutritional status (MNA-SF > 11), *n* (%)	154 (24.7)
At risk [8–11], *n* (%)	266 (42.6)
Malnutrition (< 8), *n* (%)	204 (32.7)
Barthel Index, score, median [IQR]	65 [30–100]
CPS, score, median [IQR]	2 [0–4]
CCI, score, median [IQR]	3 [2–5]
OHAT, score, median [IQR]	1 [0–3]
Reason for admission	
Infections, *n* (%)	179 (28.7)
Rehabilitation, *n* (%)	89 (14.3)
Malignancy, *n* (%)	86 (13.8)
Nutrition support, *n* (%)	82 (13.1)
Gastroenterological disorders, *n* (%)	67 (10.7)
Cardiovascular diseases, *n* (%)	22 (3.5)
Surgery, *n* (%)	21 (3.4)
Renal diseases, *n* (%)	5 (0.8)
Others, *n* (%)	73 (11.7)

Abbreviations: SD, standard deviation; BMI, body mass index; MNA-SF, mini nutritional assessment short form; IQR, interquartile range; CPS, cognitive performance scale; CCI, Charlson Comorbidity Index; OHAT, oral health assessment tool

Comparisons of the characteristics among groups based on the OHAT score (OHAT 0, OHAT 1–2, and OHAT ≥3) are presented in Table 2. Patients with an OHAT score of ≥3 are likely to be old, malnourished, inactive, and cognitively impaired as compared to those with OHAT scores of 0 and 1–2 after making post-hoc Bonferroni corrections.

**Table 2:** Comparisons of characteristics based on oral health

Variables	OHAT 0(*n* = 213)	OHAT 1–2(*n* = 206)	OHAT ≥ 3(*n* = 205)	*p* value
Age, years, mean ± SD	83.1 ± 8.0	83.1 ± 8.0	85.2 ± 7.6	0.007†‡
Male, *n* (%)	78 (36.6)	87 (42.2)	89 (43.4)	0.317
BMI, kg/m^2^, mean ± SD	20.6 ± 3.8	20.6 ± 3.9	20.0 ± 3.5	0.130
Nutrition				
Normal nutritional status (MNA-SF > 11), *n* (%)	69 (32.4)	55 (26.7)	30 (14.6)	< 0.001†‡
At risk (8–11), *n* (%)	94 (44.1)	87 (42.2)	85 (41.5)	
Malnutrition (< 8), *n* (%)	50 (23.5)	64 (31.1)	90 (43.9)	
Barthel Index, score, median [IQR]	90 [40–100]	65 [31.25–100]	45 [10–75]	< 0.001*†‡
CPS, score, median [IQR]	1 [0–3]	2 [0–4]	3 [2–5]	< 0.001*†‡
CCI, score, median [IQR]	3 [1–5]	3 [1–4]	3 [2–5]	0.021‡
Reason for admission				< 0.001*†
Infections, *n* (%)	44 (20.7)	62 (30.1)	73 (35.6)	
Rehabilitation, *n* (%)	24 (11.3)	30 (14.6)	35 (17.1)	
Malignancy, *n* (%)	43 (20.2)	23 (11.2)	20 (9.8)	
Nutrition support, *n* (%)	20 (9.4)	28 (13.6)	34 (16.6)	
Gastroenterological disorders, *n* (%)	23 (10.8)	28 (13.6)	16 (7.8)	
Cardiovascular diseases, *n* (%)	6 (2.8)	8 (3.9)	8 (3.9)	
Surgery, *n* (%)	15 (7.0)	5 (2.4)	1 (0.5)	
Renal diseases, *n* (%)	3 (1.4)	1 (0.5)	1 (0.5)	
Others, *n* (%)	35 (16.4)	21 (10.2)	17 (8.3)	

*: *p* <  0.05, OHAT 0 group vs OHAT 1–2 group using post-hoc Bonferroni correction

†: *p* <  0.05, OHAT 0 group vs OHAT ≥3 group using post-hoc Bonferroni correction

‡: *p* < 0.05, OHAT 1–2 group vs OHAT ≥3 group using post-hoc Bonferroni correction

Abbreviations: SD, standard deviation; BMI, body mass index; MNA-SF, mini nutritional assessment short form; IQR, interquartile range; CPS, cognitive performance scale; CCI, Charlson Comorbidity Index; OHAT, oral health assessment tool

**Fig. 1:**
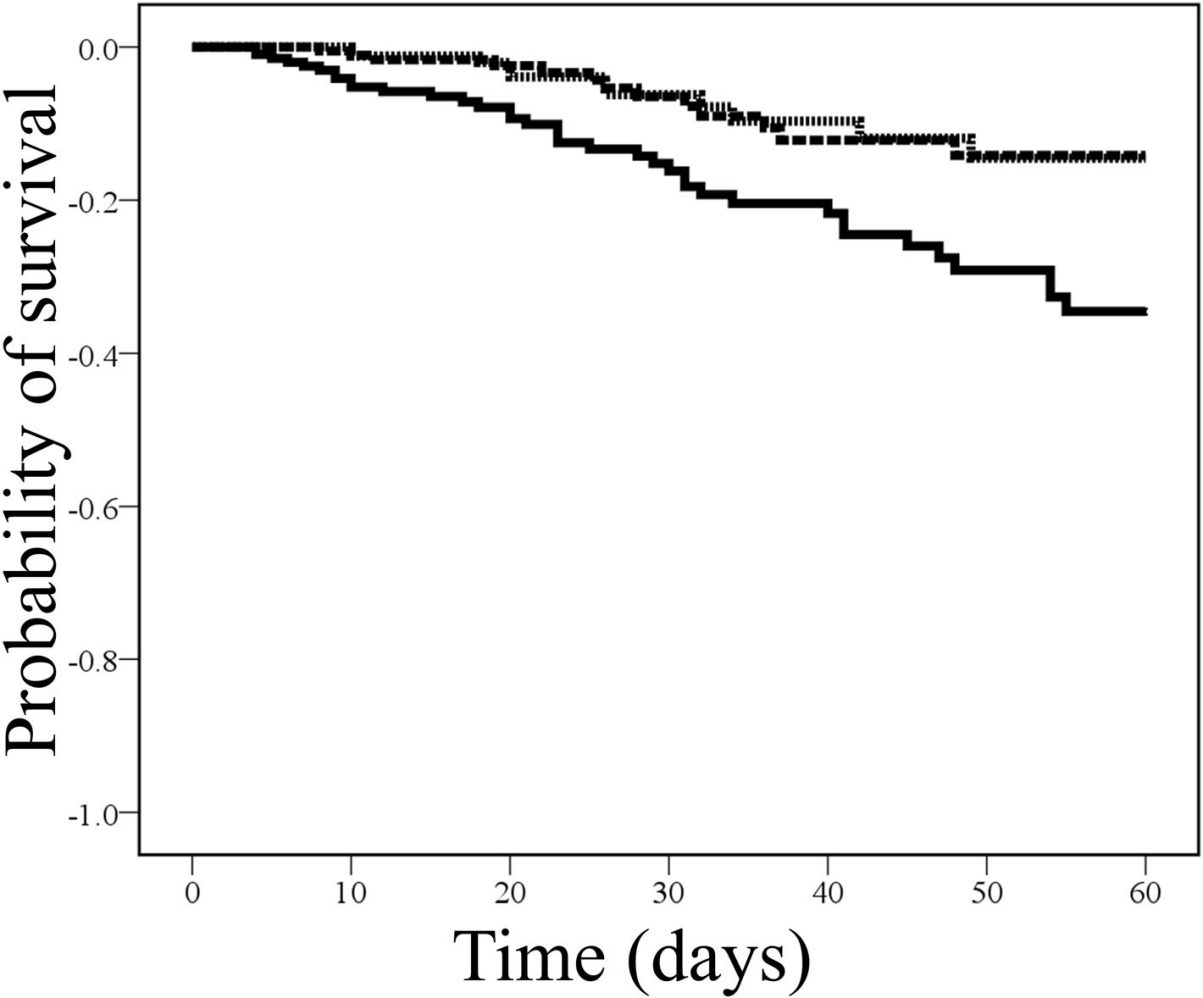
Survival curves of each group. The poorest oral health group (OHAT ≥3) showed high mortality based on log rank test after making the post-hoc Bonferroni correction in the Kaplan–Meier survival curve analysis (*p* = 0.012, OHAT 0 group vs. OHAT ≥3 group; *p* = 0.010, OHAT 1–2 group vs. OHAT ≥3 group) Solid line represents OHAT ≥3 group, and short and long dashed lines represent OHAT 0 and 1–2 groups, respectively Abbreviations: OHAT, oral health assessment tool.

Table 3 shows the outcomes of each group and statistical comparisons. The OHAT ≥3 group revealed a significantly higher mortality ratio (18.0%) as opposed to the other groups (5.2 and 6.3% in the OHAT 0 and 1–2 groups, respectively). Similar results were found when the Kaplan–Meier survival curve analysis was performed (Fig. 1). The *p* values of the log rank tests after making post-hoc Bonferroni corrections for comparisons between the OHAT ≥3 and OHAT 0 groups and between the OHAT ≥3 and OHAT 1–2 groups were 0.012 and 0.010, respectively. Furthermore, on conducting the Cox’s regression analysis for mortality after adjusting for the length of stay and other confounders, we found that patients in the OHAT ≥3 group had an independent association with mortality (hazard ratio: 2.514, 95% confidence interval: 1.220–5.183, *p* = 0.012).

**Table 3:** Comparisons of outcomes based on oral health

Variables	OHAT 0(n = 213)	OHAT 1–2(n = 206)	OHAT ≥3(n = 205)	*p* value
Mortality, *n* (%)	11 (5.2)	13 (6.3)	37 (18.0)	< 0.001*†
Length of stay, days, median [IQR]	20 [10–34]	24 [13–46]	29 [15–56]	0.001*

*: *p* < 0.05, OHAT 0 group vs OHAT ≥3 group using post-hoc Bonferroni correction

†: *p* < 0.05, OHAT 1–2 group vs OHAT ≥3 group using post-hoc Bonferroni correction

Abbreviation: OHAT, oral health assessment tool; IQR, interquartile range

## Discussion

We investigated the relationship between poor oral health and mortality in older patients admitted to our hospital in this retrospective observational study. The results of the study demonstrated the following two main findings concerning mortality and oral health: poor oral health was a predictor of in-hospital mortality and survival according to the univariate analysis as well as a multivariate analysis after adjusting for confounders; patients with poor oral health were likely to be older, malnourished, inactive, and cognitively impaired. The study was conducted using a validated tool, i.e., the OHAT score, for assessing oral health conditions.

Patients with the poorest oral health (OHAT ≥3) at hospital admission showed poor survival. To our knowledge, this is the first report involving the use of such a tool to investigate the relationship between oral health and mortality. A cohort study that enrolled military veterans demonstrated that poor oral health was an independent determinant of mortality; however, oral health in that study was diagnosed based on the subjective view of the physician after assessing the throat, mouth, teeth, gums, and tongue [18]. Moreover, such a diagnostic method was not verified for its reliability and validity. As validated tools for assessing oral health, the Revised Oral Assessment Guide (ROAG) [19] and OHAT [12] have often been used in recent studies. Poor oral health assessed as per the ROAG score demonstrated an existing association with aspiration pneumonia development [20]; however, the study did not examine the association of poor oral health with mortality. The current study was conducted with hospitalized patients. Only a few studies have investigated oral health and mortality in a hospital setting, although there are some reports, which demonstrated that oral care intervention is needed to improve oral health and the mortality rate in hospital and nursing home settings. In a systematic review, Sjögren et al. reported that mortality risk from healthcare-associated pneumonia could be reduced by oral care intervention [9]. However, how oral health at the time of intervention initiation impacts mortality remained unclear in that study.

Oral health may reflect the general condition of patients at the time of hospital admission. Our study showed that the poorest oral health at admission were related to old age, malnutrition, decreased ADL, and impaired cognition. All of these variables are considered to be related to sarcopenia [17, 21, 22], which is independently associated with poor outcomes, such as mortality, in older adults [23]. Interventions against sarcopenia such as nutritional therapy and rehabilitative exercise along with appropriate oral care intervention may reduce mortality in patients with poor oral health. A systematic review, which reported that oral care intervention alone cannot contribute toward survival of critically ill patients [24], corroborated our assumption. Future studies investigating the effect of oral care intervention in accordance with oral health at the time of its initiation on survival are expected to clarify this assumption.

There are some noteworthy limitations to our study. First, the study did not consider the severity of each disease at the time of admission, although a roughly categorized disease impact was adjusted for in the multivariate analysis. Second, although the primary outcome of the study was in-hospital death, considering time-bound mortality such as death that occurs within a certain period after admission (such as 1-month mortality) as the primary outcome would be more suitable for hospitalized patients to estimate and compare the mortality rate for a certain period. However, the retrospective design of this study did not allow us to follow up all participants for a specified period. Third, although OHAT produces a range of scores from 0 to 16, we divided the patients into 3 categories based on scores of 0, 1–2, and ≥ 3. Given that the developers of the OHAT suggest that people presenting with a score of ≥1 should be referred to a dentist [12], patients in the OHAT 1–2 group might experience unfavorable outcomes. However, in this study, we examined the association between oral health at admission and in-hospital mortality and found that the primary outcome employed in this study was not associated with the OHAT 1–2 assessed at hospitalization. Categories for which the patient’s condition is identified as ‘changes’ that affect the OHAT 1–2 score might relate to outcomes in the more distant future and/or outcomes other than mortality, such as incidence of complications, comorbidities, and further decline of oral health. Thus, studies investigating the impacts of changes in oral health on other outcomes should be considered.

## Conclusions

We provided evidence that poor oral health in older adult patients at the time of hospitalization was associated with in-hospital mortality. Studies on oral care intervention and/or other types of interventions in patients with poor oral health are warranted.

## Data Availability

The dataset analyzed during the current study is available from the corresponding author on reasonable request.

## Abbreviations

ADL: Activities of daily living
MNA-SF: Mini-Nutritional Assessment Short Form
OHAT: Oral Health Assessment Tool
ROAG: Revised Oral Assessment Guide
